# Organizational justice, trust, and identification and their effects on organizational commitment in hospital nursing staff

**DOI:** 10.1186/s12913-015-1016-8

**Published:** 2015-09-07

**Authors:** Su-Yueh Chen, Wen-Chuan Wu, Ching-Sheng Chang, Chia-Tzu Lin, Jung-Yuan Kung, Hui-Ching Weng, Yu-Tz Lin, Shu-I Lee

**Affiliations:** Division of Nursing, Department of Ophthalmology, Kaohsiung Medical University Hospital, Kaohsiung Medical University, Kaohsiung, Taiwan; Department of Ophthalmology, Kaohsiung Medical University Hospital, Kaohsiung Medical University, Kaohsiung, Taiwan; R.O.C Naval academy, Kaohsiung, Taiwan; Department of Marine Leisure Management, National Kaohsiung Marine University, Kaohsiung, Taiwan; Department of Information Management, R.O.C Naval academy, Kaohsiung, Taiwan; Institute of Gerontology, College of Medicine, National Cheng Kung University, Tainan, Taiwan; Department of Occupational Therapy, Shu-Zen Junior College of Medicine and Management, Kaohsiung, Taiwan; Department of Medical Record Administration, Kaohsiung Veterans General Hospital, Kaohsiung, Taiwan

## Abstract

**Background:**

It is of importance and urgency for hospitals to retain excellent nursing staff in order to improve patient satisfaction and hospital performance. However, it was found that simply increasing the salary is not the best method to resolve the problem of lacking nursing staff; it is necessary to focus on the impact of non-monetary factors. The delicate relationship between organizational justice, organizational trust, organizational identification, and organizational commitment requires investigation and clarification from more studies if application in nursing practice is to be expected. Therefore, this study was to investigate how the organizational justice perception could affect nurses’ organizational trust and organizational identification, and whether the organizational trust and organizational identification could encourage nurses to willingly remain in their jobs and commit themselves to the hospitals.

**Methods:**

A cross-sectional design was used. Questionnaires were distributed in 2013 to a convenience sample of 400 registered nurses in one teaching hospital in Taiwan: 392 were retrieved. Of these, 386 questionnaires were valid, which was a 96.5 % response rate. The SPSS 17.0 and Amos 17.0 (structural equation modeling) statistical software packages were used for data analysis.

**Results:**

The organizational justice perceived by nurses significantly and positively affects their organizational trust (γ_11_ = 0.49) and organizational identification (γ_21_ = 0.58). Organizational trust (β_31_ = 0.62) and organizational identification (β_32_ = 0.53) significantly and positively affect organizational commitment.

**Conclusions:**

Hospital managers can enhance the service concepts and attitudes of frontline nursing personnel by maximizing organizational justice, organizational trust and organizational identification. Nursing personnel would then be motivated to provide feedback to the attention and care provided by hospital management by demonstrating substantial improvements in their extra-role performance. Improved service concepts and attitudes would also facilitate teamwork among colleagues, boost the morale of the nursing faculty and reduce resignations and career changes.

**Electronic supplementary material:**

The online version of this article (doi:10.1186/s12913-015-1016-8) contains supplementary material, which is available to authorized users.

## Background

In most medical institutions, the nursing department is the largest department, and nursing staff comprise 40–60 % of total human resources. Nurses tend to be the most numerous component of medical teams and tend to have the longest and closest contact with patients. Therefore, nursing quality affects the overall image of a hospital and can even indirectly affect hospital operations. In recent years, the demand for nurses has increased in response to growth in the elderly population and changes in lifestyles and treatment-seeking behavior [1, 2]. However, a shortage of clinical nurses has increased workloads and extended working hours. Nurses often have low morale, which causes high turnover. This affects the overall efficiency and quality of medical service in hospitals. Therefore, the organizational commitment of nurses has become a major issue in the nursing science literature.

Through the process of observing resource allocation, decision making and interpersonal interaction in an organization, employees subjectively perceive the results and process as fair or unfair. This perception is defined as organizational justice [3, 4]. Numerous studies agree that the attitudes and behaviors of employees are affected by organizational justice. Organizational justice has long been considered essential for effective hands-on management and operations by corporate managers and executives [4, 5]. In the realm of organizational theory and organizational behavior, organizational justice has been a crucial concept and practice [4].

Trust can enhance the job satisfaction, organizational commitment, and productivity of employees [5–7]. A trusting atmosphere promotes cooperation, centralization of issues, effective communication, and information sharing and can compensate for the limited capabilities of employees [6, 7]. Organizational trust can also promote cooperation between employees and organizations, the organizational commitment of employees, and the intention of the organization to retain employees [8, 9]. The above findings confirm that organizational trust is essential for an effective organization. Therefore, organizations cannot overlook the importance of trust. Organizations must actively seek an improved understanding of trust and must implement measures to increase the trust of their employees [10].

Rosenberg and Trevino [11] indicated that an enterprise must have good credibility for a good relationship with external stakeholders. A good relationship with stakeholders then benefits its financial performance. More importantly, good credibility attracts better workers and improves the work motivation, morale, organizational commitment, and company loyalty of existing staff. Therefore, organizational identification affects widely varying organizational phenomena and organizational behavior, including decision-making, work attitudes, work motivation, job satisfaction, job performance, achievements of organizational goals, role conflict, employee interaction, staff turnover, and organizational efficiency [12].

In addition to managerial and administrative personnel, frontline nursing staff are also an important force in planning and executing organizational goals of the hospital [13]. The organizational commitment of the nursing staff depends on whether or not the staff acknowledge and accept the organizational goals or values and whether or not the staff are willing to make efforts to improve the efficiency of the hospital. Organizational commitment is reflected in the the work performance of the nursing staff and the success of the hospital in achieving organizational goals.

Mowday et al. [14] indicated that individuals have high commitment toward their organizations if they have good connections to their organizations. A high organizational commitment benefits the employee, the organization, and society. Thus, the commitment of nurses can be seen as a bridge between individual nurses and their hospital organizations. Nurses who have high commitment to their hospital organizations are willing to invest effort in their hospitals. Blegen et al. [15] analyzed factors that increase adverse events in hospitalized patients and found that, compared to patients treated in hospitals with few experienced nurses, patients treated in hospitals with numerous experienced nurses have significantly lower error rates, readmission rates, infection rates, and mortality rates and experience significantly fewer falls and pressure sores. In hospitals with many experienced nurses, patients and their families also tend to have fewer complaints. Therefore, retaining nursing staff by enhancing their organizational commitment is a matter of utmost importance and should be prioritized by hospital administrators.

Until now, no studies have focused on the relationships among organizational justice, organizational trust, organizational identification, and organizational commitment. Thus, this study searched for correlations among these factors by performing an empirical analysis from the perspective of the medical organization. Hopefully, this study can help managers in the medical service industry understand the needs of nurses and the factors that affect their job satisfaction so that they can modify or establish effective human resource systems accordingly.

## Conceptual framework

### Relationship between organizational justice and organizational trust

The equity theory proposed by Adams [16] is based on social exchange theory and extends the concept of organizational justice. Equity theory has been applied widely in the field of organizational behavior. Scholl et al. [17] defined organizational justice as the degree to which workers are cognizant that they are treated fairly in the workplace. Greenberg [18] asserted that organizational justice is the fairness of the treatment received by employees in their workplace. This treatment can serve to describe a working environment in terms of whether it is fair to employees. However, models of equity theory and distributive justice cannot entirely predict how employees react to perceived unfairness in the workplace. Studies of procedural factors that affect reward distribution have gradually increased. These studies indicate that the perceived fairness of a reward distribution is less important than the perceived procedural fairness. Therefore, studies of organizational justice have begun to shift their focus from distributive justice to procedural, i.e., the perceived justice of processes. Procedural justice is an extension of the concept of distributive justice and originates in the fields of law and politics. Thibaut and Walker [19] were the first sociologists to perform systematic studies of procedural fairness, particularly in dispute resolution. In their study of court proceedings, they defined procedural justice as the opportunity to express opinions and to participate in process control. In litigation, for example, participants likely to perceive the litigation outcome as fair if they are allowed to express their opinions and participate in the process, regardless of the whether the outcome is positive or negative. According to the perceived procedural justice theory proposed by the authors in that study, the fairness of a legal proceeding as perceived by the participants is just as important as the actual outcome. Folger and Greenberg [20] categorized organizational justice as distributive justice (the perceived fairness of the reward allocation) and procedural justice (the perceived fairness of the decision-making process applied by the organization). However, Bies and Moag [21] argued that the concepts of distributive justice and procedural justice do not adequately explain organizational justice because they do not consider the interpersonal interactions perceived by employees during procedures. Thus, they proposed the concept of interactional justice. Since then, this concept has been applied in studies of how employees in organizations perceive the fairness of their treatment and the fairness of their interpersonal communications.

Social exchange theory is applicable to trust and can be used by organizations to explain trust behavior between leaders and their employees or between an organization and its employees. Organizational trust can be considered an informal agreement between employees and their organizations or managers [22]. Trust is defined as the belief that a trustee will consider the interest of the trustor even if the trustor is not in a position to assess or obstruct negative actions by the trustee. Definitions of trust typically touch on the expectation or belief that other people will act predictably and not be entirely in their own interests [23]. Therefore, trust is defined as the belief by a trustor that others will consider the interests of the trustor even in situations where the trustor is incapable of a potentially negative response such as misunderstanding, incorrectly evaluating, or obstructing the situation. Trust is a psychological state resulting from the willingness to accept harm due to positive expectations about the intentions or behavior or others [24]. Trust is a positive expectation resulting from roles, relationships, experiences, and interdependence with others [25]. Organizational trust is a dimension that includes both horizontal and vertical factors. Horizontal trust is a relationship between peers in similar working environments. Vertical trust is the relationship that an individual employee forms with a supervisor, senior managers, or with the overall organization. Horizontal relationship trust is the willingness of an employee to accept negative consequences of organizational activities. This trust relationship is based on the expectation that the organization should not be required to monitor its employees constantly [26]. Vertical trust also includes systematic trust, which occurs when workers are sensitive and cautious about complying with the overall organizational system and attempt to understand whether the organization is worthy of their trust. Employees often carefully monitor the actions of their organization to determine whether they should trust the organization [27].

Researchers in India found that organizational trust is related to organizational justice and work performance and that organizational trust increases if employees perceive that organizational justice is adequate [28]. Since procedural justice, which is one aspect of organizational justice, is vital for developing organizational trust [29], committees that are responsible for procedural justice can positively affect organizational trust [30, 31]. Moorman et al. emphasized that, as a predictor of organizational trust, procedural justice is better than distributive justice [32]. Korsagard et al. found that procedural justice has significant positive effects on all all dimensions of organizational trust [33, 34]. Other studies have reported a positive relationship between trust and perceived fairness (justice) [35, 36]. When the organizational justice perceived by nursing staff is high, i.e., when they perceive that the hospital adequately values their contribution and supports their rights, their in the hospital and its managers increases. Table 1 lists other studies on the influence of organizational justice on organizational trust. Based on the literature, hypothesis 1 in this study was that the organizational justice perceived by nurses is positively associated with their organizational trust.

**Table 1:** Empirical studies of literature review

Researchers (Year)	Results and management findings
*Empirical studies on the influence of organizational justice on organizational trust*	
Moorman et al. [33]	This study’s findings stressed that the influence of procedural justice is more significant than that of distributive justice in predicting organizational trust.
Konovsky & Pugh [22]	Procedural justice is an important factor in employees’ citizenship behavior. Distributive justice cannot predict organizational citizenship behavior and organizational trust and support.
Korsagard et al. [34]	This study indicated that procedural justice has a greater influence on trust than distributive justice does.
Aryee et al. [13]	This study indicated that organizational justice can predict trust in and support for organizations and supervisors within organizational trust, thereby predicting organizational citizenship behavior and performance.
*Empirical studies on the influence of organizational justice on organizational identification*	
Tyler & Blader [37]	The Group Engagement Model developed in this study explains the formation of group membership. The assumptions of this model infer that perceptions of organizational justice significantly and positively influence organizational identification.
Lipponen et al. [38]	This study found that organizational justice could predict employees’ identification with their organizations.
Lipponen & Olkkonen [39]	The results of this study performed on a geographical research institute in Finland with 270 employees indicated that organizational justice (distributive justice and procedural justice) positively influenced organizational identification.
Fuchs & Edwards [45]	This study examined 137 market research employees and found that organizational justice, particularly fair interpersonal treatment of workers, positively influenced organizational identification.
Kreiner & Ashforth [46]	This study used multiple regression analysis and indicated that procedural justice can predict workers’ organizational identification in multinational companies.
*Empirical studies on the influence of organizational trust on organizational commitment*	
Nicholson & Johns [55]	This study indicated that workers with psychological contracts with high degrees of trust in their organizations have better work ethics and strong organizational commitment. In contrast, workers with psychological contracts with low degrees of trust in their organizations participate less in organizational affairs and have weak commitment. This study shows that implicit trust seems to influence organizational commitment.
McCauley & Kuhnert [56]	This study indicated that workers with strong feelings of trust in their organizations feel more satisfied with their jobs and have higher commitment toward their organizations.
Geyskens & Steenkamp [57]	This study indicated that employees’ trust in their organizations was positively associated with their organizational commitment.
Brockner et al. [59]	This study found that workers support and are committed to their management teams and organizations when their trust in their organizations’ upper management teams is high.
Schoorman et al. [60]	This study found a positive correlation between organizational trust and organizational commitment.
*Empirical studies on the influence of organizational identification on organizational commitment*	
Pratt [67]	This study indicated that organizational identification is viewed as a link between the individual and organization. This means that individuals assess the significance of their organizations toward themselves. Employees that identify with their organizations have higher organizational commitment.
Riketta [68]	This study indicated that organizational identification among workers is positively associated with attitudes toward organizational commitment.
Cole & Bruch [53]	This study found that organizational identification is seen as a part of organizational commitment. The two are highly correlated.
Knippenberg & Schie [69]	This study indicated that organizational identification is generated when the internal members of an organization have a self-defined perceptual awareness of all phenomena shaped by the organization. When organizational members are in a psychological state of identification with their organizations, relationships with the organization are further constructed. This is organizational commitment.
Van Dick et al. [70]	The results of this study indicate that organizational identification has a negative influence on employees’ turnover intentions. That is, organizational identification is positively associated with employee organizational commitment.

### Relationship between organizational justice and organizational identification

Regardless of whether employees identify with an organization, the development of organizational trust is a dynamic process that requires individuals to link themselves with social factors it doesn’t make sense [37]. Social identity theory can be used to analyze and understand the development of organizational identification. According to social identity theory, the self-concept of an individual includes a personal identity and a social identity [38, 39]. Deschamps and Devos [40] defined individual identity as the characteristics or distinct personality of an individual. That is, all individuals have unique attributes that differentiate them from others. Social identity refers to the perception of belonging to a group within a social category. The members of a group have a strong social identify if they perceive that they all have similar backgrounds. As group identity strengthens, the group begins to differentiate itself clearly from other groups. According to social identity theory, both the individual identity and the social identity of an individual are formed by constant interaction within a group [41]. For example, as an individual identity forms, interaction with a group of people who have a similar identity increases, which then results in the formation of a social identity. Social identity theory holds that people generally wish to maintain a positive self-image. Organizational identification is a special form of social identification in which individuals consider themselves members of a special group. When individuals identify with groups, they perceive that they belong to those groups [42]. Therefore, when the self-concept of an individual has characteristics resembling the characteristics perceived in the organization, organizational identification forms a cognitive connection between the individual and the organization [43].

The Group Engagement Model developed by Tyler et al. explained how members of a group form relationships [44]. The group-value model suggests that individuals tend to join groups to obtain long-term relational information and to obtain messages regarding self-value. The relationship between an individual and a group determines how strongly the individual identifies with the group [45]. This model hypothesizes that organizational justice has a significant positive effect on organizational identification [46]. Researchers have also reported a correlation between organizational justice and organizational identification [38, 44]. High organizational justice can make an individual feel respected and proud to be part of the organization. This feeling of respect and pride also enhances the identification with the organization [37]. In their study of a 270-employee geographic research organization in Finland, Lipponen and Olkkonen reported that organizational justice (distributive and procedural justice) positively affect organizational identification [39]. Table 1 shows other studies of the influence of organizational justice on organizational identification. The above studies consistently show that whether employees perceive that their organization is fair affects their identification with the organization. Therefore, hypothesis 2 was that organizational justice is positively associated with organizational identification.

### Relationship between organizational trust and organizational commitment

In The Organization Man (1956), Whyte introduced the concept of organizational commitment [47]. Another study by Becker [48] defined organizational commitment as a mechanism for ensuring continued professional conduct by the members of an organization. However, the concept and meaning of organizational commitment vary according to the perspectives of the researchers. For example, a literature review by Morrow [49] revealed over 25 different definitions of organizational commitment, which indicates the widely varying views of this concept. However, the many different definitions are essentially consistent. This line of research has remained active in the literature for many years. Commitment reflects the strength of the investment in an organization by its employees [50]. Commitment also represents the attitudes or tendencies that link the individual to the organization. These links are extremely important to the individual and valuable to the organization and overall society [51]. According to Straw [52], organizational commitment has the following characteristics: (1) it increases support for the organization by the employee; (2) it induces employees to work hard for the organization regardless of the results of their efforts; and (3) it is positively associated with individual satisfaction. According to another definition, organizational commitment results from employees having the same goals as their organization and desiring to continue their working relationships with the organization [53]. Scholars have also defined organizational commitment as the acceptance of the values of the organization and the willingness to make sacrifices [54]. That is, organizational commitment refers to the psychological state of employees who identify with the philosophy and goals of their organization. Employees who have strong organizational commitment are thus willing to continue working in the organization and are willing to work cooperatively to achieve organizational goals. This high work involvement results from the job satisfaction created by organizational identification.

Organizational trust requires a positive emotional exchange between an organization and its employees. Thus, organizational trust should correlate positively with organizational commitment. Nicholson and Johns [55] suggested that employees with high organizational trust also have a strong work ethic and a strong organizational commitment, which implies that organizational trust affects organizational commitment [56, 57]. Mirza and Redzuan [58] identified a significant positive correlation between organizational trust and commitment. Studies of marketing personnel have found that organizational trust has a strong positive impact on emotional commitment [59, 60] and therefore has a significant positive impact on organizational commitment [61].

Organizational trust is a general feeling of trust in the organization while organizational commitment is a series of behaviors exhibited by an employee after organizational trust is established. That is, organizational trust refers to thoughts whereas organizational commitment refers to action. Table 1 summarizes the findings of other studies of the influence of organizational trust on organizational commitment. The above literature suggested that nursing personnel have a strong commitment to their organization if they have high trust in the organization. Accordingly, hypothesis 3 was that the organizational trust of nursing personnel is positively associated with their organizational commitment.

### Relationship between organizational identification and organizational commitment

Employees who have a strong identification with their organization are likely to continue working for the organization and to make their best effort to benefit the organization [62, 63], i.e., they are likely to have high organizational commitment. An employee who has a strong organizational identification evaluates the organization positively and is unlikely to leave the organization [64–66]. Organizational identification reflects the general satisfaction of employees with their organization and their assessment of its image, attractiveness, and relevance. Organizational commitment refers to the relationship constructed between an individual and a group [53, 67, 68].

The relationship between an individual and a group is established by the process of organizational behavior development. For instance, many researchers agree that organizational identification is established when employees can perceive and identify with the image of their organization [69, 70]. Employees who identify with their organization develop a strong relationship with their organization, i.e., they develop organizational commitment [71, 72]. Table 1 also summarizes the results of other studies on the influence of organizational identification on organizational commitment. Based on the above literature, hypothesis 4 was that the organizational identification of nurses is positively associated with their organizational commitment.

### Research model of the theoretical relationships

**Fig. 1:**
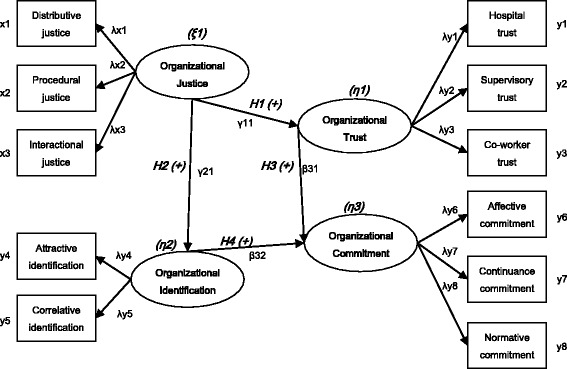
Research model of the relationships among organizational justice, organizational trust, organizational identification, and organizational commitment. *Note.* ξ(ksi): latent exogenous variables (by x), η(eta): latent endogenous variables (by y), x: observed exogenous variables, y: observed endogenous variables, λx (lambda x): coefficient of ξi and xi (ξ→x), λy (lambda y): coefficient of ηi and yi (η→y), γ(gamma): coefficient of ηi and ξi (ξ→η), β(beta): coefficient of ηi and ηj (η→η)

After an extensive literature review, this study developed a model in which organizational justice is the independent variable, organizational trust and organizational identification are the intervening variables, and organizational commitment is the dependent variable. Figure 1 is an overview of the research model.

## Methods

### Research subjects and sampling method

After receiving approval from the Hospital Institutional Review Board (IRB), a 40-item questionnaire was used to survey a convenience sample of nursing personnel employed at a single large and influential medical center in southern Taiwan. According to Chang, Chen, and Lan [1] and Chang and Chang [2, 73–75], the likelihood of identifying statistically significant findings increases as the sample size increases. However, statistical significance may not be useful from a management perspective and may yield statistical misrepresentations; thus, they suggested that, when sampling multiple departments of a hospital, the number of samples should be at least 10–15 times the number of questionnaire items (the questionnaire in this study had 40 items) and the number of samples for each unit should exceed 30 (i.e., the sample size should be sufficiently large). Therefore, this study randomly selected ten departments of the medical center. The nursing director of each selected department was asked to distribute questionnaires to 40 (i.e., more than 30) nurses in the department. The nursing director made the questionnaires available for nurses to complete anonymously onsite and ensured each respondent that completing the questionnaire was voluntary. The respondents were allowed to request clarification by the nursing director if they encountered questionnaire items that they did not understand. After completing the questionnaire survey, each respondent received a 7-Eleven gift certificate worth NT$100. To maximize the response rate, the nursing director also followed up with respondents who did not complete the questionnaire on site. Out of 400 questionnaires distributed during April-May of 2013, 386 valid and complete questionnaires were retrieved, which was a 96.5 % response rate. Therefore, the number of questionnaires analyzed in this work had a satisfactory consistency with the theoretical sample size.

## Study tools

The organizational justice scale mainly measures subjective perceptions of employees regarding organizational decision-making processes, employee incentives, performance evaluations, and interactions between managers and employees. The organizational justice scale developed by Niehoff and Moorman was modified to include the variables investigated in this study [76]. The scales developed by Hubbell and Chory-Assad [9] and by Niehoff and Moorman [76] were used for the questionnaire items. Considering the goals of this study and the cultural and industrial characteristics of the study population, a pretest was developed by three professors of nursing science, four medical experts, and seven nurses. After obtaining satisfactory results in a pilot study of 38 nurses, the questionnaire was administered in the full study population. In the principal component analysis of the sample data collected in this study, varimax orthogonal rotation revealed the same three factors reported by Niehoff and Moorman [76]: distributive justice, procedural justice, and interactional justice. Each factor had three questions. After factor analysis, distributive justice, procedural justice, and interactional justice had Cronbach α values of 0.892, 0.865, and 0.820, respectively. After rotation, the explained variances for distributive justice, procedural justice, and interactional justice were 31.616 %, 24.520 %, and 23.099 % respectively. Cumulative explained variance was 79.235 %. Because reliability refers to the reliability of the results obtained from the data measurements, the Cronback α value is generally used to test “consistency” or “stability” among items used for measuring variables. According to Nunnally [77], Cronbach α values greater than 0.70 indicate high reliability, values between 0.35 and 0.70 indicate acceptable reliability, and values lower than 0.35 indicate low reliability. Therefore, the scale in this study had extremely high reliability.

The organizational trust scale measures the concepts generated from cognition among employees, between employees and managers, and between employees and the overall organization within social exchange or cooperative relationships. This scale was revised to include the invariables investigated in this study as described in Rempel et al. [78]. The scales developed by Lenard et al. [79] and by Tzafrir et al. [80] were adopted for the questionnaire items. After revision by three professors, four medical experts, and seven nurses according to the goals of this study and the cultural and industrial characteristics of the population, a pretest was performed in 38 nurses. Finally, the questionnaire was administered in large quantities.

After deleting invalid questionnaires, factor analysis revealed three factors. Each factor had three questions. The Cronbach α values for distributive justice, procedural justice, and interactional justice were 0.897, 0.862, and 0.833, respectively, and the explained variances after rotation were 33.620 %, 25.121 %, and 24.108 %, respectively. Cumulative explained variance was 82.849 %. These statistics indicate that the reliability coefficients (Cronbach α values) for each dimensional scale exceeded the minimum value of 0.7 suggested by Nunnally [77]. Therefore, this scale also had extremely high reliability.

The scale for perceived organizational identification measures the awareness and responses of workers in specific states. This psychological state is subjective cognition that can be sufficient to link the attitudes and behaviors of the individual to the roles required by the organization. This scale was revised to form the variables to be investigated in this study with reference to Reichers [81] and Wan-Huggins et al. [82]. The Organizational Identification Questionnaire developed by Cheny and Tompkins [12] and the work of Dutton et al. [43] was used for the questionnaire items. Considering the goals of this study and the cultural and industrial characteristics of the study population, a pretest was developed by three professors of nursing science, four medical experts, and seven nurses. After obtaining satisfactory results in a pilot study of 38 nurses, the questionnaire was administered in the full study population.

Two factors emerged after factor analysis. Each factor had five questions. The Cronbach α values were 0.915 and 0.873. Explained variance values after rotation were 43.179 % and 31.956 %. Cumulative explained variance was 75.135 %. These data confirmed that the reliability coefficients (α values) for each dimensional scale exceeded the minimum of 0.7 suggested by Nunnally [77]. Therefore, this scale also had extremely high reliability.

The scale for perceived organizational commitment mainly measures a normative force generated internally by the individual. These feelings result in a sense of identification with organizations, professions, jobs, and values. This scale was revised to form the variables investigated in this study by referring to Mowday et al. [14]. The scales developed by Ho et al. [83] and by Feather and Rauter [84] were used for the questionnaire items. After revision by three professors, four medical experts, and seven nurses according to the goals of this study and the unique cultural and industrial characteristics of the study population, a pretest was performed in 38 nurses. Finally, the questionnaire was administered in large quantities. Factor analysis revealed three factors: affective commitment, continuance commitment, and normative commitment. Each factor had four questions. The Cronbach α values for affective commitment, continuance commitment, and normative commitment were 0.920, 0.886, and 0.827, respectively, and the explained variance values after rotation were 33.791 %, 26.138 %, and 24.156 % respectively. Cumulative explained variance was 84.085 %. Thus, the reliability coefficients (α values) for each dimensional scale exceeded the minimum of 0.7 suggested by Nunnally [77]. Therefore, this scale also had extremely high reliability.

The questionnaire used a 5-point Likert scale from 1 for extreme disagreement to 5 for extreme agreement. Table 2 summarizes the constructs and variables, including operational definitions for all variables. Questionnaires were examined for reliability and validity as follows:

1. Reliability analysis: Principal component factor analysis was used to extract major contributing factors, and orthogonal varimax rotation was performed to maximize the differences in factor loading carried by every common factor after the rotation to help recognize common factors. Table 3 shows the analytical results, which indicated that all Cronbach α values exceeded 0.7 [77, 85].
2. Construct convergent validity (confirmatory factor analysis): Confirmatory factor analysis can achieve higher recognition compared to expert content validity [85]. Table 4 lists the results for all dimensions. All adequacy indicators approximated the ideal values. Parameter (λ) between each latent variable and manifest variable was estimated to determine the significance of the estimated parameter (λ) in order to evaluate convergent validity. Thus, as Table 5 shows, the t values for the factor loading of all measurement items reached the significance level (*p* < 0.01), no single factor included only one question, and the composite reliability values for all constructs exceeded 0.6, which demonstrated satisfactory convergent validity [85–87].

**Table 2:** Summary of constructs and variables

Construct	Variable	Operational definition	References
Organizational justice	Distributive justice	How the nursing staff think about fairness of the resource allocation results and decisions made by the hospital.	[9, 76]
	Procedural justice	How the nursing staff think about fairness of the procedure of the decision-making standards or evaluation methods on which the hospital’s reward and punishment decisions are based.	
	Interactional justice	How the nursing staff think about fairness of the hospital’s communication with them before making any decision.	
Organizational Trust	Hospital trust	The degree to which the nursing staff trust the hospital, meaning the degree to which nursing staff, under the circumstances of trust and risk taking, are willing to trust the hospital after overall evaluation of the decisions made by the hospital.	[79, 80]
	Supervisory trust	The degree to which the nursing staff trust their supervisors, meaning the degree to which nursing staff, under the circumstances of trust and risk taking, are willing to trust their supervisors after evaluating their supervisors’ decisions and attitudes.	
	Co-worker trust	The degree to which the nursing staff trust their coworkers, meaning the degree to which the nursing staff, under the circumstances of trust and risk taking, are willing to trust their coworkers after evaluating their coworkers’ job performances.	
Organizational identification	Attractive identification	Degree of attracting nursing personnel and making them dedicate themselves to the operation of the organization.	[12, 63]
	Correlative identification	Degree of creating a close relationship with the nursing staff and making the nursing staff understand the hospital.	
Organizational commitment	Affective commitment	Degree that the nursing staff keep working at the hospital because they want to do so.	[83, 84]
	Continuance commitment	Degree that the nursing staff keep working at the hospital because they have to do so.	
	Normative commitment	Degree that the nursing staff keep working at the hospital because they are obliged to do so.

**Table 3:** Results of reliability analyses

Construct	Factor naming	Cronbach’s α (> .6)
Organizational justice		
	Distributive justice	0.892
	Procedural justice	0.865
	Interactional justice	0.820
Organizational trust		
	Hospital trust	0.897
	Supervisory trust	0.862
	Co-worker trust	0.833
Organizational identification		
	Attractive identification	0.915
	Correlative identification	0.873
Organizational commitment		
	Affective commitment	0.920
	Continuance commitment	0.886
	Normative commitment	0.827

**Table 4:** Results of convergent validity analysis

Indicator	Organizational justice	Organizational trust	Organizational identification	Organizational commitment	References
*χ*2/df. (< 3)	2.71	3.03	2.95	2.36	[86]
GFI (> .9)	0.93	0.89	0.92	0.92	
AGFI (> .8)	0.86	0.83	0.85	0.88	
NFI (> .9)	0.91	0.90	0.92	0.91	[89]
RMSR (< .08)	0.06	0.07	0.05	0.03	[91]

*Note. χ* ^2^/df. Ratio of Chi-square, *GFI* Goodness of Fit Index, *AGFI* Adjusted GFI, *NFI* Normal Fix Index, *NNFI* Non-Normal Fix Index, *CFI* Comparative Fit Index, *RMSR* Root Mean Square of Standardized Residual

**Table 5:** Confirmatory factor analysis of all the constructs

Construct	Variable/question item	Standardized loading	Measurement error	Composite reliability *(>0.6)*	AVE *(>0.5)*
Organizational justice	Distributive justice			0.82	0.77
	1. I believe my compensation is up to the standard and fair.	0.84*	0.29		
	2. Overall speaking, the rewards I received are quite fair.	0.83*	0.31		
	3. I feel that my job responsibility is fairness.	0.86*	0.26		
	Procedural justice			0.77	0.75
	4. My supervisor makes sure he/she understands every nursing staff member’s thoughts before making any decision.	0.77*	0.41		
	5. Every decision made by my supervisor can be applied to every nursing staff member affected by it.	0.82*	0.33		
	6. I can raise questions on or make appeals to the decisions made by the hospital management.	0.80*	0.36		
	Interactional justice			0.72	0.71
	7. When making my job-related decisions, my supervisor would treat me with respect.	0.69*	0.52		
	8. When making my job-related decisions, my supervisor would discuss the implication of the decision with me.	0.79*	0.38		
	9. When making my job-related decisions, my supervisor would explain the content very clearly.	0.73*	0.47		
Organizational trust	Hospital trust			0.87	0.80
	1. As far as I am concerned, most of the coworkers think that the hospital is trustworthy.	0.83*	0.31		
	2. I believe that the hospital’s promise to take care of the nursing staff is sincere.	0.85*	0.28		
	3. I believe that the hospital is fair to all nursing staff.	0.91*	0.17		
	Supervisory trust			0.83	0.76
	4. I believe my supervisors sincerely care about the nursing staff’s opinions.	0.86*	0.26		
	5. I believe my supervisors make wise decisions for the sake of the future of the hospital.	0.79*	0.38		
	6. I believe my supervisors care about nursing staff’s welfare.	0.82*	0.33		
	Co-worker trust			0.82	0.75
	7. I know that my coworkers will try their best to help me resolve the problems at work.	0.81*	0.34		
	8. I believe that my coworkers will give me a hand when I am in need.	0.76*	0.42		
	9. I am confidence in my coworkers’ job skills.	0.82*	0.33		
Organizational identification	Attractive identification			0.87	0.77
	1. In the future, I will still feel proud of being a member of this hospital.	0.79*	0.37		
	2. This hospital’s image in the community quite represents my image.	0.82*	0.32		
	3. I think that I have a strong emotional connection to this hospital.	0.86*	0.27		
	4. I usually take the hospital’s issues as my personal issues.	0.91*	0.18		
	5. As a member of this hospital, it’s my responsibility to make it more competitive.	0.80*	0.36		
	Correlative identification			0.85	0.76
	6. I am of the work location and environment of the hospital.	0.79*	0.38		
	7. The thought of continuing to work at this hospital in the future and help people makes me happy.	0.84*	0.29		
	8. I care about all the future information related to this hospital.	0.87*	0.25		
	9. My hard work can be evaluated by the overall performance of the hospital.	0.74*	0.45		
	10. Continuing to work at this hospital can improve my work capabilities.	0.78*	0.39		
Organizational commitment	Affective commitment			0.81	0.78
	1. I am glad that I am able to devote my future career life to this hospital.	0.82*	0.33		
	2. I am happy to talk about my hospital with those who are not related to the hospital.	0.84*	0.29		
	3. I am emotionally attached and belonged to this hospital.	0.55*	0.56		
	4. I strongly feel that I am part of the hospital.	0.71*	0.49		
	Continuance commitment			0.79	0.79
	5. It would be a great loss for me to quit from this hospital.	0.66*	0.56		
	6. I have the desire to keep working at this hospital at this moment.	0.71*	0.49		
	7. I think that there will be less job options for me if I leave this hospital.	0.81*	0.34		
	8. The main reason for me to stay at this hospital is that other companies won’t necessarily provide me with better compensation and benefits.	0.70*	0.51		
	Normative commitment			0.77	0.75
	9. I think it is unethical to change jobs constantly.	0.60*	0.64		
	10. The main reasons for me to keep working at this hospital are being loyal and ethical.	0.72*	0.48		
	11. I was taught to be loyal to the hospital I serve.	0.61*	0.63		
	12. I think that staying at the same hospital will have better career development.	0.81*	0.34		

*Note.* * t- value > 2; AVE = Average Variance Extracted

## Data analysis methods

The SPSS 17.0 and Amos 17.0 (structural equation modeling) statistical software packages were used for data analysis and processing, including descriptive statistical analysis to determine the sample characteristics and structural equation modeling (SEM). According to Chang and Chang [88, 89], SEM clarifies the extent of relationships between variables as well as the chain of cause and effect. Restated, SEM results do not merely show empirical relationships between variables when defining the practical situation. Therefore, SEM was used to test Hypotheses 1 to 4. Other indices used to evaluate overall model fitness were Chi-square ratio (<3), goodness of fit index (GFI > .9), adjusted goodness of fit index (AGFI > .8), normal fit index (NFI > .9) and root mean square of standardized residual (RMSR < .08) [83].

## Ethical considerations

This study was performed only after obtaining approval from the Institutional Review Board in Taiwan and written consent from all participants. Participants were assured that all personal data would remain anonymous and confidential and would be used only for research purposes. The response period was limited to two months. An introduction letter attached to the questionnaire explained the purpose of the study and ensured that all information provided by the respondent would be confidential. The questionnaire also provided an address for requesting a copy of the published study.

## Results

### Characteristics of samples

Table 5 shows the demographic data for the sample population in this study. Almost half (45.3 %) of the participants were aged 31–40 years. Most (85.0 %) participants had a bachelors degree. In terms of marital status, most (56.5 %) were married. Regarding seniority, most (41.7 %) had 10 or more years of nursing experience, and most (37.6 %) were currently assigned to the surgical department. Regarding qualifications, 58.3 % were N2 level (see Table 6).

**Table 6:** Descriptive statistics of sample (*N* = 386)

Description	Frequency	Percentage (%)
Gender		
Male	6	1.6
Female	380	98.4
Age		
30 or under	148	38.3
31–40	175	45.3
41–50	59	15.3
51 or above	4	1.1
Marriage		
Married	218	56.5
Not married	168	43.5
Education		
College or under	42	10.9
Bachelor	328	85.0
Master or above	16	4.1
Seniority		
Less than 3 years	10	2.6
3–6 years	99	25.7
6–10 years	116	30.0
10 years or above	161	41.7
Department		
Internal medicine	130	33.7
Surgical	145	37.6
Gynecology and pediatrics	73	18.9
Others	38	9.8
Job title		
N1	53	13.7
N2	225	58.3
N3	100	25.9
N4	8	2.1

### Structural equation modeling

According to the results in this study, organizational justice (including three variables: distributive justice, procedural justice, and interactional justice) had a significant positive effect on organizational trust (γ_11_ = 0.49, *p* < .01) and organizational identification (γ_21_ = 0.58, *p* < .01). The perceived distributive justice aspect of organizational justice (path coefficient, .56) had the greatest effect on organizational trust and organizational identification, and interactional justice (path coefficient, .43) had the smallest impact. Organizational trust (including three variables: hospital trust, supervisory trust, and co-worker trust) (β_31_ = 0.62, *p* < .01) and organizational identification (including two variables: attractive identification and correlative identification) (β_32_ = 0.53, *p* < .01) had significant positive effects on organizational commitment (including three variables: affective commitment, continuance commitment, and normative commitment). Additionally, organizational trust in the hospital (path coefficient, .57) and attractive identification perception of organizational identification (path coefficient, .59) had the largest impacts on organizational commitment, and co-worker trust (path coefficient, .52) and correlative identification (path coefficient, .46) had the smallest impacts. Thus, Hypotheses 1–4 and all sub-hypotheses in this study were supported (Table 7). Table 7 shows the path effects for each construct in this study. The path analysis results indicated that organizational trust and, to a lesser extent, organizational commitment had the largest direct effects on the path of organizational commitment. Organizational identification had the smallest direct effect on the path of organizational commitment. These results indicate not only that organizational trust and organizational identification directly affect organizational commitment, but also that organizational justice indirectly affects organizational commitment through organizational trust and organizational identification.

**Table 7:** Results of structural equation modeling

*Parameter estimates among latent variables*				
Path		Path name	Path coefficient	*t-*Value
Organizational Justice (ξ1) → Organizational Trust (η1) (H1)		γ11	0.49	10.77**
Organizational Justice (ξ1) → Organizational Identification (η2) (H2)		γ21	0.58	27.22**
Organizational Trust (η1) → Organizational Commitment (η3) (H3)		β31	0.62	32.35**
Organizational Identification (η2) → Organizational Commitment (η3) (H4)		β32	0.53	16.73**
*Parameter estimates among latent variables and manifest variables*				
Path		Path name	Path coefficient	*t-*Value
Organizational Justice (ξ1) → Distributive justice (x1)		λ1	0.56	21.12**
Organizational Justice (ξ1) → Procedural justice (*x*2)		λ2	0.50	11.29**
Organizational Justice (ξ1) → Interactional justice (x3)		λ3	0.43	6.81**
Organizational Trust (η1) → Hospital trust (y1)		λ4	0.57	23.03**
Organizational Trust (η1) → Supervisory trust (y2)		λ5	0.54	18.21**
Organizational Trust (η1) → Co-worker trust (y3)		λ6	0.52	14.41**
Organizational Identification (η2) → Attractive identification (y4)		λ7	0.59	29.94**
Organizational Identification (η2) → Correlative identification (y5)		λ8	0.46	9.03**
Organizational Commitment (η3) → Affective commitment (y6)		λ9	0.67	36.81**
Organizational Commitment (η3) → Continuance commitment (y7)		λ10	0.55	19.12**
Organizational Commitment (η3) → Normative commitment (y8)		λ11	0.51	13.02**
*Direct effect, indirect effect, and total effect among latent variables*				
Independent variable	Dependent variable	Direct effect	Indirect effect	Total effect
Organizational justice				
	Organizational Trust	0.49	0	0.49
	Organizational Identification	0.58	0	0.58
	Organizational Commitment	0	0.61	0.61
Organizational trust				
	Organizational Commitment	0.62	0	0.62
Organizational identification				
	Organizational Commitment	0.53	0	0.53
*Results of all the hypotheses and sub-hypotheses*				
Hypothesis and sub-Hypothesis				Result
*Hypothesis 1*: Nurses’ organizational justice has a significant positive effect on organizational trust.				Confirmed
*Hypothesis 1-1*: Nurses’ distributive justice has a significant positive effect on organizational trust.				Confirmed
*Hypothesis 1-2*: Nurses’ procedural justice has a significant positive effect on organizational trust.				Confirmed
*Hypothesis 1-3*: Nurses’ interactional justice has a significant positive effect on organizational trust.				Confirmed
*Hypothesis 2*: Nurses’ organizational justice has a significant positive effect on organizational identification.				Confirmed
*Hypothesis 2-1*: Nurses’ distributive justice has a significant positive effect on organizational identification.				Confirmed
*Hypothesis 2-2*: Nurses’ procedural justice has a significant positive effect on organizational identification.				Confirmed
*Hypothesis 2-3*: Nurses’ interactional justice has a significant positive effect on organizational identification.				Confirmed
*Hypothesis 3*: Nurses’ organizational trust has a significant positive effect on organizational commitment.				Confirmed
*Hypothesis 3-1*: Nurses’ hospital trust has a significant positive effect on organizational commitment.				Confirmed
*Hypothesis 3-2*: Nurses’ supervisory trust has a significant positive effect on organizational commitment.				Confirmed
*Hypothesis 3-3*: Nurses’ co-worker trust has a significant positive effect on organizational commitment.				Confirmed
*Hypothesis 4*: Nurses’ organizational identification has a significant positive effect on organizational commitment.				Confirmed
*Hypothesis 4-1*: Nurses’ attractive identification has a significant positive effect on organizational commitment.				Confirmed
*Hypothesis 4-2*: Nurses’ correlative identification has a significant positive effect on organizational commitment.				Confirmed

*Note.* ** *p* < .01, *χ* ^2^ / *d.f.* = 2.62, GFI = .94, AGFI = .89, NFI = .91, RMSR = .029

## Discussion

The major finding of this study is that the organizational justice perceived by nurses has a significant positive association with their organizational trust and organizational identification; additionally, organizational trust in the hospital and organizational identification are positively associated with organizational commitment. This section discusses the empirical results and provides suggestions for practitioners and subsequent studies.

## Conclusions and implications

1. The results of this study confirmed research hypothesis that the organizational justice perceived by nurses affects their organizational trust and organizational identification. The following associations were identified.
  1. Distributive justice perceived by nurses: The empirical results of this study indicate that the distributive justice perceived by nurses significantly and positively affects their organizational trust and organizational identification. Distributive justice had the greatest influence among the three variables within organizational justice. Path analysis revealed that the distributive justice perceived by nurses positively influenced organizational trust in the hospital, management trust, and coworker trust. This variable also positively influenced attractive identification and associative identification. Therefore, the empirical results were in line with our previous inferences. When decisions and actions involving the distribution of organizational resources were perceived as fair, nurses exhibited high organizational trust and high organizational identification.
  2. Perceived procedural justice: The empirical results of this study indicate that the procedural justice perceived by nurses significantly and positively affected their organizational trust and organizational identification. Path analysis revealed that the procedural justice perceived by nurse positively influenced, organizational trust in the hospital, management trust, and coworker trust. Procedural justice also had positive effects on attractive identification and associative identification. Therefore, the empirical results were consistent with our previous inferences. When nurses perceived that their organizations applied fair and clear standards for establishing employee incentives, they were likely to have high organizational trust and high organizational identification.
  3. Perceived interactional justice: The empirical results indicate that the interactional justice perceived by nurses significantly and positively affected their organizational trust and organizational identification. However, interactional justice had the least influence of the three variables within organizational justice. Path analysis also revealed that the interactional justice perceived by nurses positively affected organizational trust in the hospital, management trust, and coworker trust. Interactional justice also positively influenced attractive identification and associative identification. Therefore, the empirical results for interactional justice were also in line with our previous inferences. Nurses who perceived that their organizations made efforts to communicate with employees and elicit their opinions before making decisions were likely to have high organizational trust and organizational identification.
2. The results of this study confirmed the research hypothesis that the organizational trust perceived by nursing staff affects their organizational commitment, *i.e.,* a significant positive effect was identified. The empirical results indicate that organizational trust in hospitals, management trust, and coworker trust significantly and positively affected affective commitment, continuance commitment, and normative commitment. Of the three variables for organizational trust, organizational trust in the hospital had the largest influence whereas coworker trust had the least influence. In terms of organizational commitment, perceived organizational trust had the strongest influence on affective commitment but the weakest influence on normative commitment. Therefore, from the perspective of social exchange theory, nurses who have high organizational trust in their hospitals are likely to exhibit affective commitment.
3. The analytical results of this study confirmed the research hypothesis regarding the effects of perceived organizational identification on organizational commitment. That is, a significant positive influence was identified. The empirical results of this study of nursing personnel indicate that attractive identification and associative identification have a significant positive effect on affective commitment, continuance commitment, and normative commitment. Of the two variables of organizational identification, attractive identification had the most influence whereas associative identification had the least influence. In terms of organizational commitment, organizational identification had the largest effect on affective commitment and had the smallest effect on normative commitment. Therefore, nurses who have high attractive identification, that is, nurses who work in hospitals in which they find the image and characteristics of the hospital attractive, are likely to exhibit high work quality and high learning motivation. As a result, they are likely to display affective commitment behavior.

## Contributions to theory and practice

Traditional principles of management emphasize centralized control while overlooking the unofficial dimensions and humanities of organization. Hence, amendments to the traditional management principles emerge, of which, Elton Mayo’s Hawthorne study is the most famous. Mayo’s finding of employees’ “social psychological” factors to enhance performance by improving work (tangible and intangible) environment has highlighted the importance of human resources and organizational behavior theories in organizational management and performance [3]. In the realm of organizational theory and organizational behavior, organizational justice, trust, identification, and commitment have been the crucial concept and practice. However, the lack of existing literature on the correlation between the four factors has created a research gap in previous evidence-based practice studies on nursing personnel. Thus, this study searched for correlations among these factors by performing an empirical analysis of structural equation modeling from the perspective of the medical organization to bridge such a gap in the existing literature on nursing personnel. According to the research results, the nursing staff’s perceived organizational justice could strengthen their organizational trust and organizational identification, and further improve their organizational commitment. This kind of relationship is almost never discussed in the research literature on nursing personnel, and it can be applied for hospital human resources management to retain good nurses and reduce their turnover, the ultimate goal is to increase efficiency in the use of valuable human resources by increasing the organizational commitment of nurses, which decelerates the recruitment-training-turnover cycle in nursing staff, and thus enhancing the hospital performance.

Regarding the aforementioned human resource management activities, the following suggestions concerning practices are proposed for the reference of hospital operators, in the hope of not only helping nursing personnel to develop expertise, solve patients’ problems and achieve teamwork performance, as expected by the International Council of Nurses [90], but also create the perception of organizational justice, trust, and identification among nursing personnel, and subsequently inspire the display of their organizational commitment.

1. It goes without saying that the hospital leadership and administrative management should make efforts to improve nursing staff’s perceived organizational justice to improve their organizational commitment in the end. Therefore, when implementing policies, the hospital can start from “the results of hospital’s fair allocation on resources and compensation”, “the hospital provides equal opportunities for nursing staff to participate in decision making process and values the quality of the review process”, and “the hospital tries to eliminate the myth that the less nursing staff gets involved in the work the less workload they have”. In this way, the hospital could improve nursing staff’s perceived distributive justice, perceived interactional justice, and perceived procedural justice, and further improve nursing staff’s organizational commitment at last.
2. The results of this study indicate that the organizational commitment of nurses is affected by their organizational trust. That is, a nurse who experiences a loss of trust in a hospital has a weaker identification with the hospital, which weakens the organization. Therefore, hospitals must ensure that the nurses have adequate trust in the hospital. Following are some methods for developing nursing staff’s organizational trust perception in the hospital. First, the hospital should ensure that nursing staff are satisfied with their employee rights and benefits. Second, when nursing staff face difficulties, the hospital should strive to provide the resources needed to resolve their problems. Finally, the hospital should provide sufficient and fair support to nursing staff. The hospital must first demonstrate its trust and support for the staff in order to receive from its staff.
3. Nursing staff’s identification in the hospital affects their organizational commitment to it. Therefore, the hospital should focus on the development of nursing staff’s identification. Four suggestions are made to raise nursing staff’s high awareness on organizational identification: (1) the hospital should provide a meaningful work connection with nursing staff and that connection needs to correspond to nursing staff’s values; (2) the hospital should construct a mechanism where nursing staff can exert their expertise and accomplish their tasks, and this mechanism can elicit nursing staff’s potential; (3) the hospital should instill professional responsibility in nurses to improve the professional working relationship and communication between nursing staff and the hospital; (4) the hospital should build a system where support is always available so nursing staff won’t feel isolated and helpless at work.

## Research limitations and future studies

Finally, the findings of this study should be considered in view of the following limitations.

1. This empirical study used data obtained in a cross-sectional study. 2 months of which were spent on sampling. Inferences about the causal relationship are limited by potential sampling bias. Further studies are needed with sufficient time and funding for a vertical-section study and longitudinal analysis of the issue to obtain further verification. Further literature reviews may reveal additional moderating or mediating (intervening) variables. Future studies can also consider including additional variables that affect organizational commitment in nurses, such as role identification, work self-esteem, and leadership style. Including these variables would provide researchers with more in-depth findings.
2. The questionnaire design in this study was based mainly on academic theories, research, and the current status of the healthcare industry, and each question was designed to be comprehensive and explanatory. Likert scales were used to evaluate how nurses perceived each variable. Since all nurses surveyed in this study were currently employed by their hospitals, they may have been reluctant to make negative judgments. Additionally, the answers to the questionnaires were based on the recollection of the participants, which could have introduced bias, rather than on an objective source. Finally, the questionnaire can become inapplicable very quickly in the rapidly changing healthcare environment of today. Therefore, future studies must revise the questionnaire content according to the changing status of the healthcare environment and to meet their research needs.
3. The subjects of this study were selected from various hospital departments. Because hospitals have complex administrative systems with numerous departments, not all nursing personnel were asked to complete the questionnaire. Although the empirical research results are quite representative, they do not consider the full nursing staff. Besides, the causes and formative processes of their perceptions organizational justice, organizational trust, organizational identification, and organizational commitment could be quite subtle and complex. Future studies are recommended to use case studies for qualitative and quantitative analyses studies; this approach would provide a more comprehensive understanding of perceived organizational justice, organizational trust, organizational identification, and organizational commitment in nursing staff.
4. Although the questionnaire used as the main research instrument in this study had acceptable validity and reliability, many factors could have potentially affected the results of the voluntary survey, including defensiveness, pretending, personal emotion and other attitudes. Additionally, this study surveyed a population of nurses in only a single country. Therefore, the findings should be generalized cautiously to other populations. However, given the context of the study, the survey results exhibited adequate validity and reliability. Therefore, a similar methodology is strongly advised in future studies performed in other countries.

## Additional file

Additional file 1:
**Research Questionnaire.** (DOC 195 kb)
