# Supplementary material for: Organizational justice, trust, and identification and their effects on organizational commitment in hospital nursing staff
Source: BMC Health Serv Res. 2015 Sep 7;15:363. doi: 10.1186/s12913-015-1016-8 (PMC4562203; doi:10.1186/s12913-015-1016-8)
Supplement: Additional file 1: — Research Questionnaire. (DOC 195 kb) [file 12913_2015_1016_MOESM1_ESM.doc]

***Research Questionnaire***

**Dear Sir/Madame/Mademoiselle,**

We deeply appreciate your filling in the following questionnaire out of your tight schedule. This questionnaire is about organizational behavioural perception, aiming to understand the correlation between perception of organizational justice, organizational trust, organizational identification, and organizational commitment. It is hoped that the research finding will be valuable reference for superintendents of relevant agencies to further enhance nurses’ organizational commitment. Please read carefully the instruction of each part, and fill in the questionnaire based on your own experience. The content of the questionnaire is for academic use only, and will not be disclosed to any third party. Please feel easy to fill in this questionnaire. Again, we’d like to express our gratitude to your kind help. May you prosperity and health!

Thank you!

Yours Faithfully,

| Part One: The following are some descriptions in respect of organizational justice perception. Please fill in these questions based on your own feelings or experience. Place a check mark (🗸) in the box which best describes your personal opinions. | | | | | | | | |
| --- | --- | --- | --- | --- | --- | --- | --- | --- |
|  |  |  |  | Strongly Disagree | Disagree | Neutral | Agree | Strongly Agree |
|  |  |  |  |
|  |  |  |  |
|  |  |  |  |
|  |  |  |  |
|  | | | | | | | | |
| I. Distributive Justice | | | | | | | | |
|  |  | 1 | I believe my compensation is up to the standard and fair. | □ | □ | □ | □ | □ |
|  |  | 2 | Overall speaking, the rewards I received are quite fair. | □ | □ | □ | □ | □ |
|  |  | 3 | I feel that my job responsibility is fairness. | □ | □ | □ | □ | □ |
|  | | | | | | | | |
| II. Procedural Justice | | | | | | | | |
|  |  | 1 | My supervisor makes sure he/she understands every nursing staff member’s thoughts before making any decision. | □ | □ | □ | □ | □ |
|  |  | 2 | Every decision made by my supervisor can be applied to every nursing staff member affected by it. | □ | □ | □ | □ | □ |
|  |  | 3 | I can raise questions on or make appeals to the decisions made by the hospital management. | □ | □ | □ | □ | □ |
|  | | | | | | | | |
| III. Interactional Justice | | | | | | | | |
|  |  | 1 | When making my job-related decisions, my supervisor would treat me with respect. | □ | □ | □ | □ | □ |
|  |  | 2 | When making my job-related decisions, my supervisor would discuss the implication of the decision with me. | □ | □ | □ | □ | □ |
|  |  | 3 | When making my job-related decisions, my supervisor would explain the content very clearly. | □ | □ | □ | □ | □ |

| Part Two: The following are some descriptions in respect of organizational commitment. Please fill in these questions based on your own feelings or experience. Place a check mark (🗸) in the box which best describes your personal opinions. | | | | | | | | |
| --- | --- | --- | --- | --- | --- | --- | --- | --- |
|  |  |  |  |  |  |  |  |  |
|  |  |  |  | Strongly Disagree | Disagree | Neutral | Agree | Strongly Agree |
|  |  |  |  |
|  |  |  |  |
|  |  |  |  |
|  |  |  |  |
|  | | | | | | | | |
| I. Affective Commitment | | | | | | | | |
|  |  | 1 | I am glad that I am able to devote my future career life to this hospital. | □ | □ | □ | □ | □ |
|  |  | 2 | I am happy to talk about my hospital with those who are not related to the hospital. | □ | □ | □ | □ | □ |
|  |  | 3 | I am emotionally attached and belonged to this hospital. | □ | □ | □ | □ | □ |
|  |  | 4 | I strongly feel that I am part of the hospital. | □ | □ | □ | □ | □ |
|  | | | | | | | | |
| II. Continuance Commitment | | | | | | | | |
|  |  | 1 | It would be a great loss for me to quit from this hospital. | □ | □ | □ | □ | □ |
|  |  | 2 | I have the desire to keep working at this hospital at this moment. | □ | □ | □ | □ | □ |
|  |  | 3 | I think that there will be less job options for me if I leave this hospital. | □ | □ | □ | □ | □ |
|  |  | 4 | The main reason for me to stay at this hospital is that other companies won’t necessarily provide me with better compensation and benefits. | □ | □ | □ | □ | □ |
|  | | | | | | | | |
| III. Normative Commitment | | | | | | | | |
|  |  | 1 | I think it is unethical to change jobs constantly. | □ | □ | □ | □ | □ |
|  |  | 2 | The main reasons for me to keep working at this hospital are being loyal and ethical. | □ | □ | □ | □ | □ |
|  |  | 3 | I was taught to be loyal to the hospital I serve. | □ | □ | □ | □ | □ |
|  |  | 4 | I think that staying at the same hospital will have better career development. | □ | □ | □ | □ | □ |

| Part Three: The following are some descriptions in respect of perception of organizational trust. Please fill in these questions based on your own feelings or experience. Place a check mark (🗸) in the box which best describes your personal opinions. | | | | | | | | |
| --- | --- | --- | --- | --- | --- | --- | --- | --- |
|  |  |  |  |  |  |  |  |  |
|  |  |  |  | Strongly Disagree | Disagree | Neutral | Agree | Strongly Agree |
|  |  |  |  |
|  |  |  |  |
|  |  |  |  |
|  |  |  |  |
|  | | | | | | | | |
| I. Hospital Trust | | | | | | | | |
|  |  | 1 | As far as I am concerned, most of the coworkers think that the hospital is trustworthy. | □ | □ | □ | □ | □ |
|  |  | 2 | I believe that the hospital’s promise to take care of the nursing staff is sincere. | □ | □ | □ | □ | □ |
|  |  | 3 | I believe that the hospital is fair to all nursing staff. | □ | □ | □ | □ | □ |
|  | | | | | | | | |
| II. Supervisory Trust | | | | | | | | |
|  |  | 1 | I believe my supervisors sincerely care about the nursing staff’s opinions. | □ | □ | □ | □ | □ |
|  |  | 2 | I believe my supervisors make wise decisions for the sake of the future of the hospital. | □ | □ | □ | □ | □ |
|  |  | 3 | I believe my supervisors care about nursing staff’s welfare. | □ | □ | □ | □ | □ |
|  | | | | | | | | |
| III. Co-worker Trust | | | | | | | | |
|  |  | 1 | I know that my coworkers will try their best to help me resolve the problems at work. | □ | □ | □ | □ | □ |
|  |  | 2 | I believe that my coworkers will give me a hand when I am in need. | □ | □ | □ | □ | □ |
|  |  | 3 | I am confidence in my coworkers’ job skills. | □ | □ | □ | □ | □ |

| Part Four: The following are some descriptions in respect of organizational identification. Please fill in these questions based on your own feelings or experience. Place a check mark (🗸) in the box which best describes your personal opinions. | | | | | | | | |
| --- | --- | --- | --- | --- | --- | --- | --- | --- |
|  |  |  |  |  |  |  |  |  |
|  |  |  |  | Strongly Disagree | Disagree | Neutral | Agree | Strongly Agree |
|  |  |  |  |
|  |  |  |  |
|  |  |  |  |
|  |  |  |  |
|  | | | | | | | | |
| I. Attractive Identification | | | | | | | | |
|  |  | 1 | In the future, I will still feel proud of being a member of this hospital. | □ | □ | □ | □ | □ |
|  |  | 2 | This hospital’s image in the community quite represents my image. | □ | □ | □ | □ | □ |
|  |  | 3 | I think that I have a strong emotional connection to this hospital. | □ | □ | □ | □ | □ |
|  |  | 4 | I usually take the hospital’s issues as my personal issues. | □ | □ | □ | □ | □ |
|  |  | 5 | As a member of this hospital, it’s my responsibility to make it more competitive. | □ | □ | □ | □ | □ |
|  | | | | | | | | |
| II. Correlative Identification | | | | | | | | |
|  |  | 1 | I am of the work location and environment of the hospital. | □ | □ | □ | □ | □ |
|  |  | 2 | The thought of continuing to work at this hospital in the future and help people makes me happy. | □ | □ | □ | □ | □ |
|  |  | 3 | I care about all the future information related to this hospital. | □ | □ | □ | □ | □ |
|  |  | 4 | My hard work can be evaluated by the overall performance of the hospital. | □ | □ | □ | □ | □ |
|  |  | 5 | Continuing to work at this hospital can improve my work capabilities. | □ | □ | □ | □ | □ |
|  | | | | | | | | |

| Part Five: Personal information. | | | | | | | | | | | | | | | | | | | | | | | | | | | | | | |
| --- | --- | --- | --- | --- | --- | --- | --- | --- | --- | --- | --- | --- | --- | --- | --- | --- | --- | --- | --- | --- | --- | --- | --- | --- | --- | --- | --- | --- | --- | --- |
|  | |  | | | | | | | | | | |  | | |  | |  | | | | | | | | | | | | |
| I. | | Gender | | | | | | | | | | |  | | |  | |  | | | | | | | | | | | | |
|  | | □ | | 1. | | Male | | | | | | | □ | | | 2. | | Female | | | | | | | | | | | | |
|  | |  | | | | | | | | | | |  | | |  | |  | | | | | | | | | | | | |
| II. | | Age | | | | | | | | | | |  | | |  | |  | | | | | | | | | | | | |
|  | | □ | | 1. | | 20 years old and below | | □ | 2. | | 21-30 years old | | | | □ | | | 3. | 31-40 years old | | | | | □ | | | 4. | | 41-50 years old | |
|  | | □ | | 5. | | 51-60 years old | | □ | 6. | | 61 years old and above | | | |  | | |  |  | | | | |  | | |  | |  | |
|  | |  | |  | |  | |  |  | |  | | | |  | | |  |  | | | | |  | | |  | |  | |
| III. | | Martial Status | | | | | | | | | | | | | | | | | | | | | | | | | | | | |
|  | | □ | | (1) | | Single |  | |  |  | | | | □ | | | (2) | | Married | | | |  | |  | | |  | |  |
|  | |  | | | | | | | | | | | | | | | |  |  |  | | | | | | | | | | |
| IV. | | Education Background | | | | | | | | | | | | | | | |  |  |  | | | | | | | | | | |
|  | | □ | | (1) | | Junior College and Below | | | | | | | | | | | | □ | (2) | University | | | | | | | | | | |
|  | | □ | | (3) | | Above University (Master/ Doctor) | | | | | | | | | | | |  |  |  | | | | | | | | | | |
|  | |  | | | | | | | | | | | | | | | |  |  |  | | | | | | | | | | |
| V. | | Seniority | | | | | | | | | | | | | | | |  |  |  |  | |  | | |  | | | | |
|  | | □ | | (1) | | 3 years and less | | | | | | | | | | | | □ | (2) | 3~6 years | | | | | | | | | | |
|  | | □ | | (3) | | 6~10 years | | | | | | | | | | | | □ | (4) | More than 10 years | | | | | | | | | | |
|  | |  | |  | |  | | | | | | | | | | | |  |  |  | | | | | | | | | | |
| VI. | | Job Title | | | | | | | | | | | | | | | |  |  |  | | | | | | | | | | |
|  | | □ | | (1) | | N1 | | | | | | | | | | | | □ | (2) | N2 | | | | | | | | | | |
|  | | □ | | (3) | | N3 | | | | | | | | | | | | □ | (4) | N4 | | | | | | | | | | |
|  | |  | | | | | | | | | | | | | | | |  |  |  | | | | | | | | | | |
| VII. | | Department | | | | | | | | | | | | | | | |  |  |  | | | | | | | | | | |
|  | □ | | (1) | | Internal Medicine | |  |  |  | | □ | (2) | Surgical | | | | | |  |  | |  | | | | | | | |  |
|  | □ | | (3) | | Gynecology and pediatrics | |  |  |  | | □ | (4) | Others | | | | | |  |  | |  | | | | | | | |  |

***Many thanks for your cooperation!***
